# Development of a Score Predicting Survival after Palliative Reirradiation

**DOI:** 10.1155/2014/128240

**Published:** 2014-09-21

**Authors:** Carsten Nieder, Nicolaus Andratschke, Kent Angelo, Ellinor Haukland, Anca L. Grosu

**Affiliations:** ^1^Department of Oncology and Palliative Medicine, Nordland Hospital, 8092 Bodø, Norway; ^2^Institute of Clinical Medicine, Faculty of Health Sciences, University of Tromsø, 9019 Tromsø, Norway; ^3^Department of Radiation Oncology, University Hospital Zurich, 8091 Zurich, Switzerland; ^4^Department of Radiation Oncology, University Hospital Freiburg, 79106 Freiburg, Germany

## Abstract

*Purpose*. To develop a prognostic model for predicting survival after palliative reirradiation (PR). *Methods and Materials*. We analyzed all 87 PR courses administered at a dedicated palliative radiotherapy facility between 20.06.2007 (opening) and 31.12.2009. Uni- and multivariate survival analyses were performed, the previously published survival prediction score (SPS) was evaluated, and a PR-specific prognostic score was calculated. *Results*. In multivariate analysis, four parameters significantly influenced survival: performance status, use of steroids, presence of liver metastases, and pleural effusion. Based on these parameters, a 4-tiered score was developed. Median survival was 24.5 months for the favorable group, 9.7 and 2.8 months for the two intermediate groups, and 1.1 months for the unfavorable group (*P* = 0.019 for comparison between the two favorable groups and *P* ≤ 0.002 for all other pair-wise comparisons). All patients in the unfavorable group died within 2 months. *Conclusion*. The performance of PR-specific score was promising and might facilitate identification of patients who survive long enough to benefit from PR. It should be validated in independent patient groups, ideally from several institutions and countries.

## 1. Introduction

Palliative reirradiation is currently used in a variety of clinical settings, including but not limited to bone and brain metastases or lung and gynecological cancers [1–4]. The number of scientific publications on this topic has increased in recent years [5]. In a well-defined geographical part of Norway, palliative reirradiation contributed 10% to all palliative radiotherapy series administered during a 12-month period [6]. Randomized trials comparing single versus multiple fractions for painful bone metastases reported retreatment rates of 11–42% after a single fraction and 0–24% after multiple fractions, as summarized by Chow et al. [1]. Comparable to palliative radiotherapy in general, clinicians attempt to tailor treatment regimens to patients' prognosis, thereby minimizing undesirable over- and undertreatment. Decision aids such as prognostic scores and nomograms might facilitate rapid and reproducible assessment of patients' survival expectation by transforming the complex set of patient- and disease-related prognostic factors into a standardized tool. Ideally, prognostic scores are easy to administer and valid across different institutions and countries [7]. The Survival Prediction Score (SPS), developed and validated by Chow et al. in patient cohorts treated with palliative radiotherapy, is among the tools that might be widely applicable, because it is based on three readily available parameters: primary cancer type, site of metastases, and performance status [8]. Its performance has never been tested specifically in patients undergoing palliative reirradiation. Together with a large number of other baseline factors potentially impacting survival, we analyzed SPS in a single-institution cohort study.

## 2. Methods

We retrospectively reviewed the records of all consecutive patients who received palliative reirradiation at a single hospital with dedicated palliative radiotherapy unit. The patients started their treatment in the time period from June 20, 2007 (date of opening of the dedicated palliative radiotherapy unit), to December 31, 2009. Reirradiation was defined as partial or complete field overlap (examples of partial overlap: initial course included thoracic vertebrae Th4-6 and reirradiation Th6-8; initial course of radical prostate radiotherapy followed by pelvic bone metastasis irradiation). A total of 87 reirradiation courses were studied. Stereotactic radiotherapy was unavailable and thus not included in the present series. All medical records, treatment details, and information on date of death were available in the hospital's electronic patient record (EPR) system. The survival status and date of death or last follow-up of the patients were obtained from the EPR. Patients who were lost to follow-up were censored on the date of last documented contact (personal appointment, telephone conversation, and blood test). Median follow-up for all surviving/censored patients was 5.4 months. Survival time was measured from the start of reirradiation. Actuarial survival curves were generated by Kaplan-Meier method and compared by log-rank test (analyses performed with IBM SPSS Statistics 20). Multivariate analyses were performed by Cox regression (backward conditional method). We assigned SPS as described by Chow et al. [8], that is, based on three variables (nonbreast cancer, metastases other than bone, and Karnofsky performance status (KPS) ≤ 60): poor prognosis group when all three were present, intermediate prognosis group when two were present, and good prognosis group when 0-1 were present. Our own prognostic scores were developed as previously described by Rades et al. [9, 10]. In brief, the score for each predictive factor was determined by dividing the actuarial death rate at prespecified time points (given as the percentage) by 10. For example, patients with good KPS were assigned 0 points and those with poor KPS 1.5 points (rate of death at 1 month (15%) divided by 10). The total score represented the sum of the scores for each predictive factor. Two time points reflecting poor prognosis or short survival were chosen, 1 month and 2 months, because there is no generally agreed definition of sufficient survival expectation, justifying initiation of palliative radiotherapy. Given that recent research and discussions focused on overtreatment, for example, use of radiation therapy in the last 30 days of life, we felt that predicting short survival might be more important [11–14].

## 3. Results

**Figure 1:**
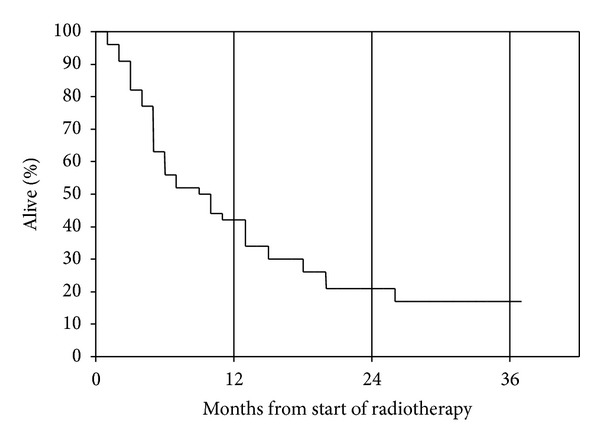
Actuarial overall survival after palliative reirradiation (Kaplan-Meier estimate).

Median age at the time of reirradiation was 67 years (range 38–90 years). Prostate (29%) and non-small cell lung cancer (NSCLC, 11%) were the most common primary tumors. Additional baseline information is shown in Table 1. Bone metastases were the prevailing target for reirradiation. The most common regime consisted of 10 fractions of 3 Gy (43%). Other common regimes included 8 Gy single fraction (uncomplicated bone metastases) and 5 fractions of 4 Gy (various sites and indications). Five courses (6%) remained incomplete, typically because of earlier than expected clinical deterioration. Median survival of this small group of patients was 2.8 months. Overall median survival from reirradiation was 8.6 months and 1-year survival rate 42% (Figure 1). Six percent of patients received radiotherapy during the final month of life. Seventeen percent of patients died within 2 months.

**Figure 2:**
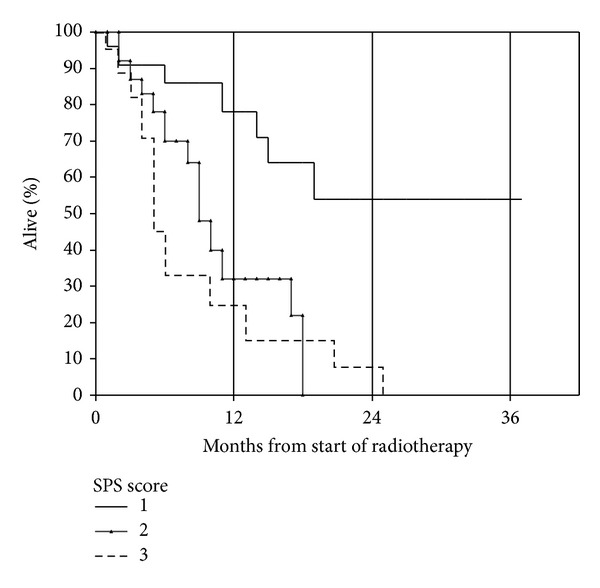
Actuarial overall survival after palliative reirradiation stratified by SPS score (Kaplan-Meier estimate): group 1 (*n* = 23), median not reached; group 2 (*n* = 26), median 6.7 months; group 3 (*n* = 38), median 4.1 months; *P* = 0.26 for group 2 versus 3 and *P* < 0.05 for other comparisons.

**Figure 3:**
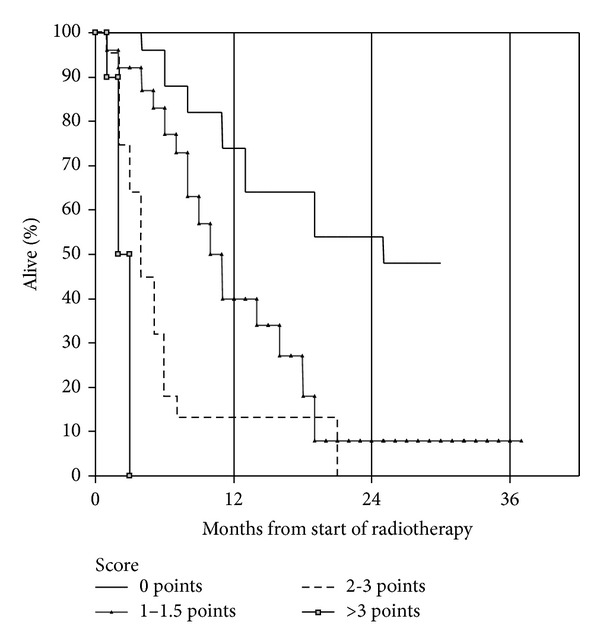
Actuarial overall survival after palliative reirradiation stratified by new score, variant 1 (Kaplan-Meier estimate): median 24.5 (0 points) versus 9.7 (1–1.5 points) versus 2.8 (2-3 points) versus 1.1 months (>3 points), *P* = 0.024 for comparison between group 1 and 2, *P* ≤ 0.003 for all other pair-wise comparisons. Number of patients in each group: 20, 24, 20, and 6. Missing information to assign score in 17 patients.

**Figure 4:**
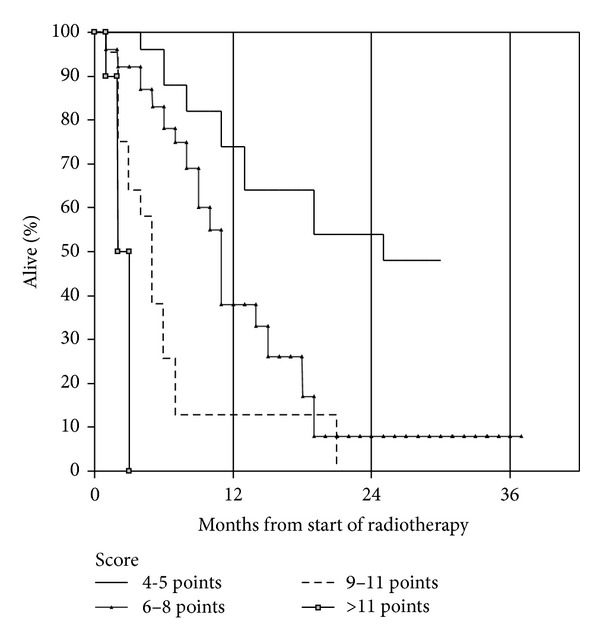
Actuarial overall survival after palliative reirradiation stratified by new score, variant 2 (Kaplan-Meier estimate): median 24.5 (4-5 points) versus 9.7 (6–8 points) versus 2.8 (9–11 points) versus 1.1 months (>11 points), *P* = 0.019 for comparison between group 1 and 2, *P* ≤ 0.002 for all other pair-wise comparisons. Number of patients in each group: 20, 26, 18, and 6. Missing information to assign score in 17 patients.

We analyzed the potential prognostic impact of all baseline parameters shown in Table 1 and assigned SPS score. However, the performance of this score was unsatisfactory because two of the three patient groups had similar survival (Figure 2). As shown in Table 2, two components of the SPS score (metastases location and performance status) significantly influenced survival, while primary tumor type did not. In multivariate analysis, a total of four parameters significantly influenced survival: KPS, use of steroids, presence of liver metastases, and pleural effusion. Based on these parameters, a new 4-tiered prognostic score was developed. As described in Section 2, we compared two different variants, which are shown in Table 3. When applying a short-survival-definition of 1 month (variant 1), the resulting survival curves separated clearly (Figure 3). Median survival was 24.5 months for the favorable group, 9.7 and 2.8 months for the intermediate groups, and 1.1 months for the unfavorable group (*P* = 0.024 for comparison between the two favorable groups and *P* ≤ 0.003 for all other pair-wise comparisons). Thirty-three percent of patients in the unfavorable group died within 1 month and all within 2 months. When applying a short-survival-definition of 2 months (variant 2), the resulting survival curves separated equally clear (Figure 4). Median survival was exactly the same as in variant 1 (*P* = 0.019 for comparison between the two favorable groups and *P* ≤ 0.002 for all other pair-wise comparisons). Since the unfavorable group included exactly the same patients, 33% died within 1 month and all within 2 months. Because of its superior significance level, variant 2 might be the preferred assignment method.

## 4. Discussion

Palliative reirradiation is an important treatment option, providing symptom improvement in many patients with bone metastases [1] and other conditions [15]. While most previous studies were small and often retrospective, the randomized bone metastases study by Chow et al. comparing different fractionation regimens included 850 patients [1]. Median survival in the two arms was 9.3 and 9.7 months, respectively. This result is comparable to the 8.6 months reported in our own, bone metastases-dominated study. However, survival of individual patients might be as short as few days or as long as several years (Figure 1). Therefore, prognostic scores might be valuable decision aids when prescribing palliative reirradiation. Chow et al. have previously published several reports on a score for patients receiving palliative radiotherapy in general, the SPS. Development of this prediction model started in 395 patients referred to their palliative radiotherapy program [16]. Later, they refined their original six-parameter-model by reducing the number of variables to three (primary cancer type, site of metastases, and performance status), arriving at the SPS [8, 17]. We hypothesized that this score might also predict survival of patients receiving reirradiation but discovered that further studies, which also include other models, are needed. The performance of the SPS score (Figure 2) can be explained by the fact that not all adverse SPS features (nonbreast cancer, metastases other than bone, and poor performance status) influenced prognosis of reirradiated patients. In the present study, metastases location and performance status significantly influenced survival, while primary tumor type did not.

Disadvantages of our study include its retrospective design and limited number of patients, especially regarding subgroups. Not all patients had complete information on all baseline parameters recorded in the EPR system. The majority of reirradiation courses consisted of hypofractionated regimens, mostly 1–15 fractions, with dose/fractionation parameters reflecting a patient's expected prognosis (clinical estimate). We did not use any particular prognostic models or scores when assigning treatment regime during the time period covered in our study. Nevertheless, more than 90% of patients who were offered reirradiation also completed their treatment. Only 6% were treated during the final month of life, suggesting that our clinical decision making was largely successful, even if further improvement should be attempted.

Our score based on KPS, use of steroids, presence of liver metastases, and pleural effusion performed promisingly. To the best of our knowledge, no other scores related specifically to palliative reirradiation exist. One of the clinical aims of applying prognostic scores might be avoidance of overtreatment in patients with very short survival [18]. Recently, Tanvetyanon et al. have reported on use of radiotherapy in the last 30 days of life in the United States [19]. They used a SEER-Medicare linked database to obtain a large study cohort of 202,299 patients who died as a result of lung, breast, prostate, colorectal, and pancreas cancers (top five cancer causes of death) between January 1, 2000, and December 31, 2007. The rate of radiotherapy in the last 30 days of life, by many regarded as inappropriate overtreatment, though this point of view is controversial, was 7.6%. No data on reirradiation were reported in this study, and no attempt was made to develop predictive models. Before our new score can be widely implemented, external validation is necessary. In the future, it might become possible to study narrowly defined patient groups, if sufficiently large databases can be created. For example, Tanvetyanon et al. have published prognostic factors for survival after salvage reirradiation in patients with head and neck cancer [19]. Rades et al. have developed scores specific to metastatic spinal cord compression [20, 21], and Sperduto et al. to brain metastases [22], both related to first line treatment rather than reirradiation.

## 5. Conclusions

Prognostic factors for survival might change during the course of disease, for example, from first line to subsequent treatments. The performance of the newly developed score was promising and might facilitate identification of patients who survive long enough to benefit from palliative reirradiation. It should be validated in independent patient groups, ideally from several institutions and countries.

## Figures and Tables

**Table 1:** Patient characteristics.

Characteristic	No.	%
Entire cohort	87	
Gender		
Male	65	75
Female	22	25
Family status^1^		
Single	20	23
Married	55	63
Partner	5	6
Missing information	7	8
Karnofsky performance status		
90–100	31	36
70–80	30	34
≤60	26	30
Primary tumor site		
Prostate	25	29
Breast	9	10
Lung (non-small cell)	10	11
Colorectal	8	9
Bladder	5	6
Kidney	6	7
Skin (malignant melanoma)	3	3
Other	21	24
Dose/fractionation (intention-to-treat)		
10 fractions of 3 Gy	24	28
Single fraction of 8 Gy	19	22
5 fractions of 4 Gy	15	17
12–15 fractions of 2.5 Gy	4	5
Other	25	29
Reirradiation target types		
Bone metastases	69	79
Brain metastases	5	6
Lung metastases or primary tumor	6	7
Other	7	8
Known brain metastases		
No	80	92
One or more	7	8
Known liver metastases		
No	68	78
One or more	19	22
Known lung metastases		
No	65	75
One or more	22	25
Known adrenal gland metastases		
No	76	87
One or more	11	13
Known bone metastases		
No	14	16
One or more	73	84
Metastatic spinal cord compression		
No	80	92
Yes (radiologic or symptomatic)	7	8
Pleural effusion		
No	81	93
Yes (radiologic or symptomatic)	6	7
Number of metastatic sites		
0	10	11
1 (e.g., lungs only)	37	43
2 (e.g., lungs and brain)	27	31
3	11	13
4	2	2
Progressive disease outside RT target volume^1^		
No	27	31
Yes	55	63
Missing information	5	6
Systemic cancer treatment^1^		
No	23	26
Within 4 weeks before RT	21	24
Within 3 months before RT	14	16
Earlier	17	20
Missing information	12	14
Use of opioid analgetics at start of RT^1^		
No	21	24
Yes	54	62
Missing information	12	14
Use of steroids at start of RT^1^		
No	32	37
Yes	38	44
Missing information	17	20
Serum hemoglobin^1^		
Low^2^	66	76
Normal	16	18
Missing information	5	6
Serum albumin^1^		
Low^2^	17	20
Normal	42	48
Missing information	28	32
Serum lactate dehydrogenase^1^		
Normal^2^	14	16
Elevated	35	40
Missing information	38	44
Serum alkaline phosphatase^1^		
Normal^2^	25	29
Elevated	29	33
Missing information	33	38
Serum creatinine^1^		
Low^2^	13	15
Normal	48	55
Elevated	15	17
Missing information	11	13
Serum C-reactive protein^1^		
Normal^2^	20	23
Elevated but less than 30 mg/L	27	31
Elevated 30–60 mg/L	14	16
Elevated >60 mg/L	17	20
Missing information	9	10
Thrombocyte count^1^		
Low^2^	11	13
Normal	45	52
High	19	22
Missing information	12	14
Charlson comorbidity index^1^		
0	7	8
1-2	44	51
3 or more	28	32
Missing information	8	9
Smoking status^1^		
Current smoker	34	39
No	34	39
Missing information	19	22

RT: radiotherapy.

^1^Missing information in some cases.

^2^Hematology and blood chemistry results refer to institutional limits of normal; only test results obtained within one week before RT were considered.

**Table 2:** Prognostic factors for survival. All baseline variables shown in Table 1 were analyzed (univariate, log-rank test). Those with *P* value <0.1 were carried forward to multivariate Cox regression analysis and are shown here.

Characteristic	Median survival (months)	P value	
		Univariate^1^	Multivariate
Karnofsky PS			
90–100	18.3	0.0001	0.0001
70–80	9.4		
≤60	2.1		
Known brain metastases			
No	9.7	0.008	n.s.
Yes	3.6		
Known liver metastases			
No	9.7	0.037	0.039
Yes	2.8		
Pleural effusion			
No	9.4	0.007	0.039
Yes	1.3		
Number of metastatic sites			
Max. 2	9.7	0.054	n.s.
3 or more	2.8		
Progressive disease outside RT target volume			
No	12.6	0.033	n.s.
Yes	5.5		
Use of opioid analgetics			
No	24.5	0.02	n.s.
Yes	5.2		
Use of steroids			
No	12.2	0.002	0.015
Yes	3.6		
Serum albumin			
Low	9.7	0.001	n.s.
Normal	2.8		
Serum alkaline phosphatase			
Normal	15.1	0.027	n.s.
Elevated	4.1		
Serum creatinine			
Low	1.6	0.0001	n.s.
Normal	9.7		
Elevated	15.1		
Serum C-reactive protein			
Normal	18.3	0.0001	n.s.
Elevated but less than 30 mg/L	12.6		
Elevated 30–60 mg/L	5.3		
Elevated >60 mg/L	2.6		
Thrombocyte count			
Low	12.7	0.038	n.s.
Normal	9.7		
High	4.0		
Number of abnormal blood tests^2^			
Max. 1	12.7		
2	5.8	0.008	n.s.
3 or more	3.0		
Smoking status			
Current smoker	4.3	0.063	n.s.
No	9.7		
Time from first cancer diagnosis			
Shorter than median (47 months)	5.3	0.089	n.s.
Longer than median	9.7		

RT: radiotherapy; PS: performance status.

^1^If more than 2 groups, *P* value from log-rank test pooled over all strata.

^2^All tests shown in Table 1 were considered.

Significance levels were not corrected for multiple tests.

**Table 3:** Prognostic scores based on four parameters predicting survival in multivariate analysis. Endpoints: death within 1 month (variant 1) and death within 2 months (variant 2).

Parameter	Died within 1 month	Points^1^	Died within 2 months	Points^1^
Karnofsky PS				
70–100	2%	0	7%	1
≤60	15%	1.5	39%	4
Known liver metastases				
No	4%	0	8%	1
Yes	11%	1	49%	5
Pleural effusion				
No	4%	0	14%	1
Yes	33%	3	50%	5
Use of steroids				
No	3%	0	10%	1
Yes	11%	1	28%	3
Minimum sum score		0		4
Maximum sum score		6.5		17

PS: performance status.

^1^Death rate divided by 10.
